# Therapeutic Antibodies for the Treatment of Pancreatic Cancer

**DOI:** 10.1100/tsw.2010.103

**Published:** 2010-06-15

**Authors:** Patrick Chames, Brigitte Kerfelec, Daniel Baty

**Affiliations:** INSERM U624, GDR2352, Marseille Cedex, France

**Keywords:** antibodies, therapy, pancreatic, bispecific, clinical trials

## Abstract

Pancreatic cancer is a devastating disease with the worst mortality rate and an overall 5-year survival rate lower than 5%. In the U.S., this disease is the fourth leading cause of death and represents 6% of all cancer-related deaths. Gemcitabine, the current standard first-line treatment, offers marginal benefits to patients in terms of symptom control and prolongation of life. Since 1996, about 20 randomized phase III trials have been performed to improve the efficacy of gemcitabine, with little success regarding a significant improvement in survival outcomes. The need for novel therapeutic strategies, such as target therapy, is obvious. Monoclonal antibodies have finally come of age as therapeutics and several molecules are now approved for cancer therapies. This review aims to give a general view on the clinical results obtained so far by antibodies for the treatment of pancreatic cancer and describes the most promising avenues toward a significant improvement in the treatment of this frustrating disease.

### INTRODUCTION

#### Pancreatic Cancer

Pancreatic cancer is a devastating disease with the worst mortality rate and an overall 5-year survival rate lower than 5%. Although accounting for only 3% of all cancers, this disease is the fourth leading cause of death and represents 6% of all cancer-related deaths. In the U.S., the overall incidence is about eight to ten cases per 100,000 persons/year and rises slowly over the years[1]. Pancreatic cancer remains one of the most difficult to treat due to late initial diagnosis and to intrinsic resistance to conventional treatments. About 50% of patients have distant disease at the time of diagnosis (locally advanced stage) and in 40%, the tumor has spread (metastatic stage). Risk factors have been identified[2], molecular pathogenesis has been elucidated, but advances in early detection and efficient treatments remain rather disappointing despite tremendous efforts. The majority of pancreatic tumors (95%) is adenocarcinomas and they mainly develop from exocrine cells. They are characterized by an aggressive behavior with a fast progression rate that makes them highly metastatic, by a dense fibrotic stroma surrounding a minor population of cancerous cells, and by an early and vascular invasive growth. This reactive stroma is composed of extracellular matrix proteins (collagen, fibronectin), myofibroblastic pancreatic stellate cells, immune cells, cytokines, growth factors, proteases, and vessels.

### Current Treatments

There is consensus on the fact that surgical removal of the tumor represents the best option for pancreatic cancer treatment; to be resectable, tumors need to be small and strictly localized to the pancreas without invasion into surrounding organs and evidence of metastasis. However, only 15–20% of all patients are candidates for potentially curative surgery. Depending on the tumor localization, pancreaticoduodenectomy, distal or total pancreatectomy can be performed. However, even with an optimal curative surgery, metastases often occur. Median survival time without evidence of recurrent disease is 21.2 months after resection[3].

For locally advanced or metastatic disease, treatment is still palliative rather than curative, and chemotherapy remains the only option. Since its approval in 1997, gemcitabine is the current standard first-line treatment. It was shown by Burris and colleagues[4] to improve median disease-free survival and overall survival (OS) rate at 5 years. Since 1996, about 20 randomized phase III trials have been performed to improve the efficacy of gemcitabine, with little success regarding a significant improvement in survival outcomes (for review, see [5,6,7]). A small clinical benefit is evidenced for the erlotinib/gemcitabine and platinum/gemcitabine combinations, but it must be balanced by increased toxicity. Up to now, the only combination approved by U.S. Food and Drug Administration (FDA) is the erlotinib/gemcitabine combination (Tarceva®), which increased the median OS from 6 to 6.4 months[8,9].

### New Potential Targets

Advances in the understanding of pancreatic cancer biology and of the mechanisms by which it eludes conventional cytotoxic therapies should lead to the identification of new potential targets. Cancer stem cells (CSC) might be one of them. Tumors are constituted by differentiated cells that have limited proliferative potential, and by a small subset of cancer cells responsible for tumor initiation and propagation[10,11]. In pancreatic cancer, CSC represent 0.2–0.8% of all cancer cells and they are characterized by cell surface markers CD44, CD24, and ESA (epithelial specific antigen)[12]. CSC have been shown to be resistant to conventional chemotherapy and radiation[13], which might explain the poor therapeutic results of current therapies. The contribution of tumor/stroma interactions in pancreatic cancer progression has also been recently emphasized[14,15]. Promising results in a phase II trial have been obtained by combining gemcitabine with nab-paclitaxel[16]. This compound, which is an antimitotic agent, increased the potency of gemcitabine by altering the stroma surrounding the tumor.

Thus, the need for novel therapeutic strategies, such as target therapy, in pancreatic cancer patients is obvious. Recent years have seen the approval of several therapeutic antibodies for cancer therapy (Table 1), some of them leading to very significant therapeutic effects[17]. Disappointments met with the first murine therapeutic molecules that were rapidly eliminated by the patient's immune system and that were not interacting properly with human receptors of the patient's effector cells have been replaced with significant clinical outcomes achieved by chimeric (human IgG bearing murine-variable domains), humanized (human IgG bearing murine-hypervariable loops), and fully human antibodies[17]. Therapeutic antibodies have been shown to rely on various modes of action, including direct blocking of a ligand or a receptor, induction of apoptosis by receptor clustering, complement-dependent cytotoxicity, or cell-mediated cytotoxicity/phagocytosis. The last generation of therapeutic antibodies has been engineered to interact strongly with activating receptors, such as FcγRIIIA expressed on NK cells. Modifications have targeted some residues of the Fc fragment involved in the interaction with Fc receptors, or the nature of the Fc glycosylation shown to influence greatly the affinity of the receptors for the IgG. More recently, bispecific antibodies capable of simultaneously binding to a tumor antigen and to CD3 on T cells, and thereby redirecting polyclonal T cells to tumor cells, have led to exciting clinical results[18,19].

**TABLE 1**
**Antibodies Approved for Therapeutic Use in Cancer**

| Generic Name | Trade Name | Antibody Format | Antigen | Approved Indication | FDA Approval[9] | EMEA Approval[9] |
|---|---|---|---|---|---|---|
| Rituximab[1] | Mabthera | Chimeric, IgG1 | CD20 | CD20-positive B-cell non-Hodgkin's lymphoma | 11/26/97 | 06/02/98 |
| Trastuzumab | Herceptin | Humanized, IgG1 | HER2/Neu | Metastatic breast cancer | 09/25/98 | 08/28/00 |
| Gemtuzumab[2] | Mylotarg | Humanized, IgG4 | CD33 | CD33-positive acute myeloid leukemia | 05/17/00 | NA |
| Alemtuzumab[3] | Mabcampath | Humanized, IgG1 | CD52 | B-cell chronic lymphocytic leukemia | 05/07/01 | 07/06/01 |
| Ibritomomab[4] | Zevalin $^{90}$Y | Mouse, IgG1 | CD20 | B-cell non-Hodgkin's lymphoma | 02/19/02 | 01/16/04 |
| Tositumomab[4,5] | Bexxar $^{131}$I | Mouse, IgG2a | CD20 | CD20-positive B-cell non-Hodgkin's lymphoma | 06/27/03 | NA |
| Cetuximab | Erbitux | Chimeric, IgG1 | EGFR | Metastatic colorectal and head and neck carcinoma | 02/12/04 | 06/29/04 |
| Bevacizumab[6] | Avastin | Humanized, IgG1 | VEGF-A | Metastatic colon and rectum and non–small cell lung cancer | 02/26/04 | 01/12/05 |
| Panitumumab[7] | Vectibis | Human, IgG2 | EGFR | Metastatic colorectal carcinoma | 09/27/06 | 12/19/07 |
| Catumaxomab[8] | Removab | Mouse,IgG2a/rat IgG2b | EpCAM/CD3 | Malignant ascites | NA | 05/13/09 |
| Ofatumumab[7] | Arzerra | Human, IgG1 | CD20 | B-cell chronic lymphocytic leukemia | 10/26/09 | NA |

1 Rituximab is commercialized under the trade name Rituxan in the U.S.

2 Gemtuzumab "ozogamicine" is coupled to calicheamicin, an antitumoral antibiotic.

3 Alemtuzumab is commercialized under the trade name Campath in the U.S.

4 Ibritomomab "tiuxetan" and tositumomab are coupled to radioisotopes.

5 All approved antibodies have a κ light chain except tositumomab, which has a λ light chain.

6 Bevacizumab has also been recently approved for breast (February, 2008) and renal (July, 2009) cancers, and glioblastoma (May, 2009).

7 Human antibodies obtained from humanized mice.

8 Catumaxomab is a bispecific antibody obtained from mouse and rat monoclonal antibodies.

9 Month/Day/Year; NA, not approved; EMEA, European Medicines Agency.

The aim of this paper is to review the most recent studies involving the use of antibodies for the treatment of pancreatic cancer.

## CLINICAL TRIALS INVOLVING ANTIBODIES

### Angiogenesis

#### *Vascular Endothelial Growth Factor (VEGF)*

Angiogenesis provides the required substrates for tumor growth and dissemination. Targeting angiogenesis is attractive because, in theory, it can reduce cancer progression. VEGF-A (or VEGF) is the predominant member of the VEGF family. VEGF receptor is similar to EGFR because after binding to its ligand, VEGF receptor forms a dimer, and the tyrosine kinase becomes autophosphorylated and amplifies the initial signal by activating the intracellular pathways, such as MAPK and PI3K[20]. VEGF exerts most of its neoangiogenic effects through VEGF-R2[21]. VEGF is expressed in 88–93% of the patients with pancreatic adenocarcinoma[22]. Moreover, VEGF presence in tumor samples correlates with tumor size and is also a predictor of early recurrence after surgery, development of liver metastasis, poor prognosis, and cancer-related death[23].

Bevacizumab is a recombinant, humanized, anti-VEGF-A, monoclonal antibody that prevents binding to VEGF-R1 and VEGF-R2 (Table 1). This antibody has proven clinical benefit in metastatic colon, breast, and non–small cell lung cancer. Bevacizumab decreases lymphangiogenesis and neovascularization *in vivo* and is expected to increase the delivery of chemotherapy to the tumor in pancreatic cancer. Kindler and colleagues reported the results of a phase II trial of bevacizumab plus gemcitabine in 52 patients with metastatic pancreatic cancer. Partial responses were observed in 21% of patients, the median survival time was 8.8 months, and the 1-year survival rate was 29%[24]. The major side effect with bevacizumab therapy in pancreatic cancer was bleeding and perforation. Therefore, it was not recommended in patients with tumors invading the duodenum or other organs. These data prompted the CALGB to conduct a double-blind, placebo-controlled, randomized phase III trial (CALGB 80303) of gemcitabine plus bevacizumab vs. gemcitabine plus placebo in 602 advanced pancreatic cancer patients. Gemcitabine was given in the standard fashion and bevacizumab was given at a dose of 10 mg/kg on days 1 and 15 of each 28-day cycle. Unexpectedly, the median OS times were 5.7 months and 6.0 months for gemcitabine plus bevacizumab and gemcitabine plus placebo, respectively[25]. Finally, a phase III trial of bevacizumab in combination with gemcitabine and erlotinib involving 607 patients with metastatic pancreatic cancer demonstrated that the addition of bevacizumab to gemcitabine/erlotinib does not lead to a statistically significant improvement in OS in patients, although progression-free survival (PFS) was significantly longer in the bevacizumab group compared with placebo[26].

Unfortunately, it appears that bevacizumab does not lead to clinical benefits for pancreatic cancer treatment, probably because other proangiogenic factors, including insulin-like growth factor-I, hepatocyte growth factor, fibroblast growth factor, platelet-derived growth factor, and TGF-α, are often overexpressed by pancreatic cancer cells.

#### *Integrin α5β1*

Integrins form a superfamily of widely expressed transmembrane glycoprotein receptors for extracellular matrix ligands, such as fibronectin, vitronectin, laminin, collagens, and other plasma membrane proteins, and are involved in the regulation of a broad variety of cellular processes, including embryogenesis, inflammation, bone metabolism, apoptosis, cell proliferation, angiogenesis, and tumor metastasis. Integrin α5β1, the principal fibronectin receptor, is an important survival factor that plays a key role in angiogenesis. Endothelial cell expression of the α5β1 integrin and its ligand fibronectin are both upregulated during tumor angiogenesis[27]. Volociximab, a high-affinity IgG4 chimeric monoclonal antibody, has led to a very efficient apoptosis of vascular endothelial cells, angiogenesis, and tumor growth inhibition in preclinical studies, independently of the VEGF pathway[28]. Volociximab is currently being tested in a phase II study in combination with gemcitabine in 40 patients with metastatic pancreatic cancer. Preliminary results (ASCO 2008 Gastrointestinal Cancers Symposium, abstract 142) indicate three partial remissions and 17 stable diseases. The treatment was well tolerated and these results are thus encouraging.

### Tumor Targeting

#### *Epidermal Growth Factor Receptor (EGFR)*

The EGFR, also known as ErbB1 or HER1, is a 170-kDa protein belonging to the four-member ErbB1-4 family of transmembrane tyrosine kinase growth factor receptors. Upon binding to a number of ligands, conformational alterations occur in the single-chain receptor, allowing dimerization or oligomerization with other EGFR molecules or other members of the ErbB family, leading to autophosphorylation and signal transduction. EGFR is expressed in 30–89% of pancreatic cancers assayed by immunohistochemistry techniques[29,30].

Cetuximab is an anti-EGFR therapeutic antibody already approved for the treatment of colorectal cancer (Table 1). This antibody has also been investigated in pancreatic cancer in combination with the standard gemcitabine agent. In a phase II study with chemotherapy-naïve patients with advanced disease, 41 patients with EGFR immunostain-positive tumors (six locally advanced, 35 metastatic) were treated with cetuximab (initial dose 400 mg/m$^2$, followed by 250 mg/m$^2$ weekly) and gemcitabine. Five patients (12.5%) achieved a partial response and 26 patients (63.4%) had stable disease. The median time to progression was 3.8 months and the median OS duration was 7.1 months. The 1-year survival rate of 31.7% compared favorably to 18% with gemcitabine alone in historical series. The combination was well tolerated, with the most commonly reported toxicities of all grades as follows: rash (87.8%), nausea (61.0%), weight loss (58.5%), and diarrhea (53.7%)[31]. This small, but significant, improvement warranted further studies and two trials were launched. The SWOG S0205 study was a randomized phase III trial of gemcitabine with or without cetuximab in 766 patients with locally advanced or metastatic pancreatic adenocarcinoma. The data, presented in 2007, did not evidence a statistically significant difference in PFS (3 vs. 3.5 months, $p$ = 0.14) and OS duration (6 vs. 6.5 months, $p$ = 0.058), in the gemcitabine alone vs. gemcitabine and cetuximab groups, respectively[32]. The second study (ECOG) was a randomized phase II trial in which 87 eligible patients with metastatic pancreatic cancer were randomized to the Murren regimen of docetaxel and irinotecan with or without cetuximab. Unexpectedly, the patients treated with cetuximab had shorter median survival duration (5.3 months) compared to the placebo group (6.5 months). A very short PFS was observed in the cetuximab arm, not well explained by the data. The toxicity of this regimen was high, even without cetuximab. Grade 3 or 4 diarrhea was observed in 47% of the patients in the three-drug arm and the rate of treatment-related death was 4%[33]. Thus, unfortunately, no significant improvements could be demonstrated using cetuximab. Of note, another anti-EGFR antibody, matuzumab, has demonstrated some activity in clinical trials. A phase I study assessed the safety and potential benefit of combined treatment with matuzumab and standard-dose gemcitabine[34]. Seventeen chemotherapy-naive advanced pancreatic adenocarcinoma patients received escalating doses of matuzumab (400 mg weekly, 800 mg biweekly, or 800 mg weekly) and gemcitabine. Severe treatment-related toxicities were limited to grade 3 neutropenia, leucopenia, and decreased white blood cell count. Common study drug-related adverse events were skin toxicities and fever. Matuzumab inhibited phosphorylated EGFR and affected receptor-dependent signaling and transduction; effects were seen even in the lowest-dose group. Partial response (PR) or stable disease occurred in eight of 12 evaluated patients (66.7%), with three PRs among six evaluated patients in the group receiving 800 mg weekly. Matuzumab was thus well tolerated and the combination may have enhanced activity.

Panitumumab is a fully human, anti-EGFR that prevents EGF and TGF-α from binding to the receptor (Table 1). This antibody has yielded exciting preclinical data, including the complete eradication of established human tumors (derived from the human vulvar epidermoid carcinoma cell line A431) as large as 1.2 $cm^3$[35]. A phase II clinical trial comparing gemcitabine plus erlotinib with or without panitumumab is currently ongoing in the U.S. (NCT00550836).

### Human Epidermal Growth Factor Receptor 2 (HER2)

HER2 (or ErbB2) is another member of the EGFR family. HER2 is involved in signal transduction pathways leading to cell growth and differentiation, and is overexpressed in a number of cancers, including breast and pancreatic cancer. Up to 26% of pancreatic tumor specimens express HER2 as shown by immunohistochemistry[36]. Trastuzumab is a neutralizing, humanized antibody directed against the extracellular domain of the HER2 receptor (Table 1). The therapeutic effect of trastuzumab is well documented in breast cancer, and has been ascribed to cell cycle arrest and induction of apoptosis, as well as induction of antibody-dependent cellular cytotoxicity (ADCC) against HER2-overexpressing tumor cells[37]. Based on this rationale and on several encouraging preclinical results, a phase I clinical trial enrolling 34 patients with metastasized HER2 overexpressing (grade 2–3) pancreatic cancer was launched. A combination of trastuzumab and gemcitabine resulted in partial response rates of 6%, and 41% of patients showed a >50% reduction of the tumor marker CA19-9[38]. A modest improvement of the median survival in patients with metastatic pancreatic cancer suggested that there may be a modest benefit of trastuzumab treatment for some patients. A larger phase II trial combining trastuzumab and capecitabine should start soon[39].

Interestingly, a combination of anti-HER1 and anti-HER2 antibodies was tested in preclinical studies, and demonstrated a significant improvement of survival and tumor regression in mice treated with trastuzumab plus cetuximab in first- and second-line treatments, compared with gemcitabine. These results warrant further clinical trials[40].

### Mesothelin

Mesothelin is a differentiation antigen whose expression in normal human tissues is limited to mesothelial cells lining the pleura, pericardium, and peritoneum[41]. The mesothelin gene encodes a precursor protein of 71 kDa that is processed to a 31-kDa shed protein called megakaryocyte potentiating factor (MPF) and a 40-kDa fragment, mesothelin, that is attached to the cell membrane by a glycosyl-phosphatidylinositol (GPI) anchor. Mesothelin binds the mucine MUC-16, also called CA-125[42], but little is known about its function. It has been shown that mesothelin is highly expressed in several human cancers, including virtually all mesotheliomas and pancreatic adenocarcinomas, and approximately 70% of ovarian cancers and 50% of lung adenocarcinomas. Argani and colleagues were the first to show mesothelin expression in pancreatic ductal adenocarcinoma, but not in normal pancreas[43]. In addition, mesothelin mRNA expression was present in four of four resected primary pancreatic cancers and all 60 resected primary adenocarcinomas were mesothelin positive as shown by immunohistochemistry. These results were confirmed by Hassan and colleagues who showed that mesothelin was expressed in all 18 cases of pancreatic adenocarcinomas examined, but absent in normal pancreas and in chronic pancreatitis[44]. Mesothelin is also highly expressed in other adenocarcinomas of the biliary tree, such as gallbladder cancer and tumors of the common bile duct[45]. This tumor marker is being evaluated for targeted therapy.

SS1P is a recombinant immunotoxin consisting of a mouse antimesothelin disulfide-stabilized Fv linked to a truncated *Pseudomonas* exotoxin (dsFv-PE38) that mediates cell killing upon internalization. Preclinical studies have shown that SS1P is cytotoxic to cell lines expressing mesothelin and causes complete regression of mesothelin-expressing tumor xenografts in nude mice[46]. This construct was further tested in a phase I clinical trial. Out of the 34 patients treated (20 mesothelioma, 12 ovarian cancer, and two pancreatic cancer), four had minor response and 19 had stable disease, including complete resolution of ascites in two patients[47]. The immunotoxin was well tolerated and led to some clinical results, warranting further clinical trials.

MORAb-009 is a chimeric (mouse/human) IgG1 with high affinity and specificity for mesothelin. The heavy- and light-chain variable regions of mouse antimesothelin scFv (obtained by panning on mesothelin-positive cells a phage display library made from an immunized mouse) were grafted with human IgG1 and κ constant regions. MORAb-009 was shown to prevent adhesion of mesothelin-bearing tumor cells to MUC16-positive cells and to elicit cell-mediated cytotoxicity on mesothelin-bearing tumor cells. Treatment that included MORAb-009 in combination with chemotherapy led to a marked reduction in tumor growth in nude mice compared to chemotherapy or MORAb-009 treatment alone[48]. This antibody is now being evaluated in clinical trials. A phase I study of MORab-009 on 24 mesothelioma, pancreatic, and ovarian cancer patients gave encouraging results. MORAb-009 was well tolerated and six subjects demonstrated stable disease warranting additional therapy[49]. Given the favorable safety profile and possibility of clinical benefit, a phase II study of the efficacy of MORAb-009 with gemcitabine in unresectable pancreatic cancer is underway.

### *Carcinoembryonic Antigen (CEA, CEACAM5)*

Carcinoembryonic antigen (CEA), a glycoprotein, is a tumor-associated antigen and elevated levels are detected in the cell membrane of tumors derived from the epithelium. CEA is overexpressed in over 90% of pancreatic cancers[50]. Binding of antibodies to CEA has been shown to inhibit cell migration, cell invasion, and cell adhesion, and to have antimetastatic effect *in vivo*[51]. In a phase I/II clinical trial, Sultana and colleagues enrolled 25 patients to assess the safety and efficacy of $I^{131}$-labeled, anti-CEA, chimeric (sheep/human), antibody KAb201 in patients with unresectable pancreatic adenocarcinoma[52]. In this approach based on the use of a monoclonal antibodies conjugated with radionuclides, the radiation component has a bystander effect, i.e., killing of adjacent unbound cells, while lessening toxicity to normal tissues. The results demonstrated tumor targeting, with hematological toxicity of varying degrees. Survival and efficacy data were comparable to the median survival and efficacy seen with single-agent gemcitabine. An antigenic response directed to the chimeric antibody was observed and might constitute a limitation for repeated dosing.

### *Insulin-Like Growth Factor 1 Receptor (IGF-1R)*

Cixutumumab (IMC-A12) is a fully human, monoclonal antibody (IgG1) against type 1 insulin like growth factor receptor (IGF-1R). The IGF-1R and its ligands IGF-1 and IGF-2 have been implicated as playing key roles in the development, maintenance, and progression of cancer[53]. IGF-1R signaling is known to activate both the PI3K/Akt and the MAPK pathways, leading to increased tumor cell survival and proliferation, respectively. By blocking this signaling, cixutumumab has demonstrated encouraging results in preclinical studies, with significant growth inhibition of breast, renal, and pancreatic tumors, and a marked increase in apoptotic tumor cells in antibody-treated animals[54]. A phase I/II clinical trial is currently comparing the efficiency of gemcitabine and erlotinib with or without cixutumumab for the treatment of patients with metastatic pancreatic cancer (NCT00617708).

AMG 479 is also a fully human, monoclonal, IgG1 antibody that binds IGF-1R without cross-reacting with the closely related insulin receptor. AMG 479 has been shown to inhibit binding of IGF-1 and IGF-2 to the receptor[55]. As a single agent, AMG 479 strongly inhibited the growth of pancreatic carcinoma xenografts, and long-term treatment was associated with reduced IGF-IR signaling activity and expression. The combination of AMG 479 with gemcitabine resulted in additive inhibitory activity both *in vitro* and *in vivo*. A phase II study of AMG 479 as single therapy in advanced carcinoid and pancreatic neuroendocrine tumors is currently ongoing (NCT01024387).

### PRECLINICAL STUDIES

Several other tumor targets have been explored as markers for antibody therapy of pancreatic cancer.

#### Nonspecific Cross-Reacting Antigen (NCA, CEACAM6)

CEACAM6 (CD66c) is an integral member of the CEA family. It is a cell-surface glycoprotein composed of an extracellular region containing three immunoglobulin-like domains (344 residues) and linked to the plasma membrane via a glycophosphoinositol anchor. CEACAM6 is capable of homophilic and/or heterophilic adhesion to other CEACAM family members[56]. CEACAM6 is expressed on normal human epithelial and myeloid cells, but the level of expression is 1 to 2 logs lower compared with expression in malignant tissue[57]. Several gene-expression profiling studies on pancreatic ductal adenocarcinoma (PDA) cell lines[58,59] and human PDA biopsy samples[60,61] have shown a 20- to 25-fold overexpression of CEACAM6 compared with normal pancreatic ductal epithelial cells[58,61]. CEACAM6 is also overexpressed in several other epithelial carcinomas (colon, breast, ovarian and non–small cell lung cancers)[57]. Based on these data, CEACAM6 appears as an attractive pancreatic tumor marker. However, expression of CEACAM6 protein has been noted in a variety of normal human tissues, including granulocytes, and antigen-dependent toxicity cannot be easily tested in the preclinical model since rodents do not express CEACAM6. A recent interesting preclinical study has evaluated the potential of an immunoconjugate between a chimeric (mouse/human) anti-CEACAM6 IgG and an antimitotic agent, maytansinoid (DM1), in Cynomolgus macaques[62]. The authors first confirmed the expression of CEACAM6 in >90% of invasive pancreatic adenocarcinomas as well as in intraepithelial neoplastic lesions by tissue macroarrays, and obtained a marked tumor growth inhibition in xenografted mice. As expected, the treatment induced neutropenia in macaques as a result of antigen-dependent toxicity. However, this phenomenon was fully reversible and the authors observed a repopulation from the pool of CEACAM6-negative, lineage-committed progenitors in a time span shorter than typically seen after bone marrow ablation in the course of conventional, nontargeted, cytotoxic chemotherapy. Beside this rapid, but reversible, neutrophil depletion, no adverse effects were seen, which encourages further studies of this immunoconjugate.

CEACAM6 has also been targeted in another preclinical study for the treatment of pancreatic ductal adenocarcinoma. The authors produced a humanized, anti-CEACAM6 scFv fragment and PEGylated the fragment through the introduction of a cysteine in the linker to increase serum half-life[63]. *In vivo*, the fragment inhibits tumor growth in a murine xenograft model through inhibition of angiogenesis and proliferation. It was shown to induce targeted tumor cell apoptosis specifically, without dependence on antibody-dependent cellular cytotoxicity, complement-dependent cytotoxicity, or conjugation with a cytotoxic agent. The proposed mode of action was high-affinity binding to CEACAM6, which enhances the disruption of domain-1 homophilic dimer with consequent functional inhibition of CEACAM6, known to play an important role in apoptosis resistance in conditions where cells lose contact with the extracellular matrix[64]. These promising results justify clinical trials.

#### Epithelial Cell Adhesion Molecule (EpCAM)

EpCAM is a glycosylated, 30- to 40-kDa, type I membrane protein, which is expressed in a variety of human epithelial tissues, cancers, and progenitor and stem cells. EpCAM is comprised of an extracellular domain with EGF- and thyroglobulin repeat-like domains, a single transmembrane domain, and a short 26-amino-acid intracellular domain. EpCAM in normal cells is predominantly located in intercellular spaces where epithelial cells form very tight junctions. It has, therefore, been speculated that EpCAM on normal epithelia is sequestered and, therefore, much less accessible to antibodies than EpCAM in cancer tissue, where it is homogeneously distributed on the cancer cell surface.

Moreover, EpCAM is expressed on essentially all human adenocarcinomas, on certain squamous cell carcinomas, on retinoblastomas, and on hepatocellular carcinomas[65]. Importantly, EpCAM is part of the signature of cancer-propagating cells in numerous solid tumors and of normal progenitor and stem cells[11,66]. Moreover, EpCAM is apparently needed to maintain distinct cancer cell attributes[67] and, potentially, cancer stem cell phenotypes as well.

Recently, several studies using various formats of bispecific antibodies (bsAbs) to retarget T cells to tumor cells have yielded outstanding results in cancer therapy studies[18,19]. Interestingly, several of these molecules target EpCAM. Catumaxomab, an EpCAM x CD3 bsAb, represents the first success of this new class of therapeutic antibodies as it was recently approved for the intraperitoneal treatment of patients with EpCAM-positive malignant ascites[68]. In clinical trials, this molecule could lead to very clear clinical results, including a reduction of EpCAM-positive malignant cells in ascites by up to 5 logs[69] and the induction of a long-term anticancer immunity[70]. The efficiency of this new molecule for pancreatic cancer should soon be assessed.

MT-110 is another EpCAM x CD3 bsAb that can induce potent redirected lysis of target antigen-expressing cells at pico- to femtomolar concentrations, which is accompanied by highly conditional T-cell activation. Redirected lysis involves formation of a cytolytic synapse, possibility of serial lysis at very low effector-to-target (E:T) ratios, and no longer depends on MHC class I expression or costimulatory molecules[71,72]. This molecule is also expected to be tested soon for pancreatic cancer treatment.

Finally, a third EpCAM x CD3 bsAb is yielding promising results for pancreatic cancer treatment. HEA125 x OKT3, a hybrid-hybridoma-derived murine bsAb, has recently been investigated in preclinical studies using BxPC-3 pancreatic carcinoma xenografts in nonobese diabetic (NOD) severe combined immunodeficiency (SCID) mice[73]. The bsAb could significantly retard growth of xenografts *in vivo*, and was shown to increase duration of the contact between migrating lymphocytes and tumor cells, and to stimulate secretion of effector cytokines from nonstimulated peripheral blood mononuclear cells (PBMCs) in the presence of EpCAM-bearing tumor cells *in vitro*.

### Mucin 1 (MUC-1)

MUC-1 is a mucin expressed by >85% of invasive pancreatic adenocarcinomas, including early stage I disease and the precursor lesions, pancreatic intraepithelial neoplasia, and intraductal papillary mucinous neoplasia[74]. PAM-4, a murine anti-MUC-1 IgG, targets an epitope of MUC-1, which is absent from normal and inflamed pancreatic tissues, as well as from most other malignant tissues[75]. Preclinical studies demonstrated that PAM-4 was able to target pancreatic cancer with high specificity, achieving high concentrations at the tumor site using xenografted athymic nude mice. More recently, TF10, an anti-MUC-1 x HSG bsAb, was derived from this monoclonal antibody. This bsAb can be used in pretargeted approaches in which the bsAb is first injected and is allowed to accumulate in the tumor before injecting, in a second step, a radiolabeled hapten ($^{111}$In-HSG) that is captured by the bsAb at the tumor site. This method can lead to very high tumor-to-normal tissue contrasts. When compared with its parental monoclonal antibody in studies conducted in nude mice bearing CaPan1 human pancreatic cancer xenografts, TF10 led to much greater tumor/blood ratios of $^{111}$In-HSG (1,000:1 at 3 h) compared to $^{111}$In-PAM4-IgG (5:1 at 24 h)[76]. The high therapeutic potential of this approach should thus soon be tested in the clinic.

### Death Receptor 5 (TRAIL-R2)

Tumor necrosis factor–related apoptosis-inducing ligand (TRAIL) binds to death receptors 4 and 5 (DR4, DR5) to transduce apoptotic signals. TRAIL receptors are increased in the membrane of cancer cells. More than 50% of cancer cell lines tested *in vitro* are TRAIL susceptible. Brain, lung, pancreatic, liver, ovary, rectum, cervix, testes, thyroid, stomach, and laryngopharynx cancers frequently overexpress DR4 and DR5[77]. However, the ability of soluble human TRAIL to induce apoptosis of normal human hepatocytes *in vitro* has raised questions regarding the potential usefulness of this agent in cancer therapy. TRA-8, an antihuman DR5 monoclonal antibody, induces apoptosis of most TRAIL-sensitive tumor cells both *in vitro* and *in vivo*. However, unlike TRAIL, it does not induce apoptosis in normal hepatocytes[77]. In animal models, TRA-8 produced synergistic cytotoxicity in combination with gemcitabine or irinotecan through enhanced caspase activation[78,79,80]. These findings, with substantial inhibition of tumor growth in a mouse pancreatic cancer xenograft model receiving combination therapy, are encouraging for antideath receptor therapy in the treatment of pancreatic cancer. Tigatuzumab, the humanized, IgG1 monoclonal antibody derived from TRA-8, is in ongoing phase I clinical trials at the moment[81].

Conatumumab (AMG 655) is a fully human, monoclonal agonist antibody (IgG1) to human DR5. This monoclonal antibody can also induce apoptosis via caspase activation and could inhibit tumor growth in colon (Colo205 and HCT-15), lung (H2122), and pancreatic (MiaPaCa2/T2) xenograft models[82]. These results suggest that conatumumab is a potential therapeutic agent for treating patients with multiple tumor types, including pancreatic tumors. Clinical trials are expected for soon.

#### Other Targets of Interest

Human Claudin-4 (CLDN4) is a tetraspanin transmembrane protein consisting of 209 amino acids (aa) belonging to the CLDN family, which play an important role in tight junction formation and function. The protein structure consists of cytoplasmic N- and C-termini, four transmembrane domains, and two extracellular loops. Immunohistochemical analysis and differential expression studies between normal and cancerous tissues indicate that CLDN4 expression is highly detectable in a variety of carcinomas, including pancreatic cancers. However, CLDN4 protein is detectable in normal breast, prostate, bladder, and gastrointestinal mucosa, although its expression is substantially less intense than that seen in cancer tissue samples[83]. Recently a chimeric (mouse/human) anti-CLDN4 has been evaluated in preclinical studies[84]. The monoclonal antibody KM3934 induced dose-dependent antibody-dependent cellular cytotoxicity and complement-dependent cytotoxicity *in vitro*, and significantly inhibited tumor growth in CFPAC-1 xenografted SCID mice, indicating that CDLN4 might be a valuable pancreatic tumor target.

Fetoacinar pancreatic protein (FAPP) is present at the cell surface of human pancreatic tumoral tissues. A murine antibody, mAb16D10, recognizing the O-glycosylated COOH-terminal domain of FAPP, has been developed. In a recent study[85], this monoclonal antibody stained 22 tumoral tissues over the 22 tested, independently of the tissue pretreatment (frozen sections or formalin-fixed, paraffin-embedded sections) and presented a unique specificity for membranes of neoplastic cells. Furthermore, the mAb16D10 did not react with all nonpancreatic tumoral and normal tissues tested. Moreover, intraperitoneal injections of mAb16D10 in mice xenotransplanted with human pancreatic tumoral SOJ-6 cells decreased the growth rate of the established tumor[86], indicating that this target might be of interest for antibody-based pancreatic cancer therapy.

### CONCLUSION

There is an obvious need for new therapies in pancreatic cancers. Therapeutic antibodies have been successful in a number of malignancies, but results obtained on pancreatic cancer treatments have so far been extremely frustrating, with all phase III clinical trials leading to failure. However, recent years have seen the emergence of new antibody formats, such as bispecific antibodies and the discovery of highly specific tumor markers. These advances should yield major improvements in a near future and should ultimately greatly impact the treatment of this devastating disease.
